# An Editorial View on the Special Issue “Colorectal Cancers: From Present Problems to Future Solutions”

**DOI:** 10.3390/cancers14040975

**Published:** 2022-02-15

**Authors:** Heike Allgayer

**Affiliations:** Department of Experimental Surgery—Cancer Metastasis, Mannheim Medical Faculty, Ruprecht Karls University of Heidelberg, 68167 Mannheim, Germany; heike.allgayer@medma.uni-heidelberg.de; Tel.: +49-(0)621-383-71630 or +49-(0)621-383-71635; Fax: +49-(0)621-383-71631

**Keywords:** colorectal cancer, (molecular) carcinogenesis, cancer progression, metastasis, (single) cancer cell heterogeneity, models, infectious agents, (targeted) therapy, personalized medicine

Colorectal cancer (CRC) represents one of the most frequent human cancer entities and is still amongst the “top killers” in human cancer, although fundamental progress has been made in recent years in CRC prevention, early diagnosis, basic and translational research, and (targeted) therapy. The current Special Issue, “Colorectal cancers: from present problems to future solutions”, presents 13 highly timely articles, 9 original articles and 4 reviews, which give insights into, and highlight, the latest developments within the scientific, translational and clinical CRC community, presenting views and work of several internationally highly recognized experts in the field. To this end, the special issue covers exciting novel discoveries in basic and mechanistic research that help to understand CRC carcinogenesis, progression and metastasis, tumor cell heterogeneity, and novel microenvironmental components in CRC. It also covers clinical parameters that modify CRC characteristics and therapy response, and tools and models with a high innovative potential to open new chapters in the differential diagnosis of CRC heterogeneity and response to therapy. 

Several articles present advances or novel, in part provocative, hypotheses on causes of CRC carcinogenesis, progression, metastasis, and/or CRC interaction with the (micro-) environment. In an exciting review, Nobel Laureate Harald zur Hausen and Ethel-Michele de Villiers give an intriguing overview on a potential, completely novel class of infectious agents, which might open new avenues to our understanding of indirect carcinogenesis, but also further chronic diseases, such as type 2 diabetes, as (co-) caused by bovine meat and milk factors (BMMFs) [1]. BMMFs represent a recently discovered class of infectious species with self-replicating capacity, isolated by the authors from bovine milk and meat, the structure of BMMFs being rather plasmid-like. Initial data from the authors suggest that BMMFs, taken up by nutrition, give rise to chronic inflammation and immune stimulation, thereby contributing to a rather unspecific and indirect means of local (CRC) carcinogenesis but, over and above, to a systemic priming of inflammatory contributions to the development of further malignant tumor and chronic diseases, e.g., type 2 diabetes. This discovery certainly not only widens our understanding of carcinogenesis and chronic disease in general, but bears the potential to revolutionize views on human nutrition, in particular on the milk and meat industry. Adding to potential inflammatory or infectious (co-)factors of CRC carcinogenesis or progression, a review of our own group, together with nutritional experts, discusses novel hypotheses on bacteriophages and their potential contributions to colorectal (chronic) inflammatory conditions and CRC, as modulated by particular components of human nutrition and an associated priming of intestinal microbial microenvironments [2]. Adding to novel microenvironmental insights into CRC, in the currently largest case number study of CRC patients, an exciting original article by Sjöblom and his group [3] systematically analyzes the spatial immune landscape of multiplexed CRC tissue immunofluorescence panels. The article shows exciting changes in the type of immune cells, representing both adaptive and innate immunity, between different types of CRC. To this end, CRCs of the right colon show an enrichment of most T-cell types and M2 macrophages, whereas rectal cancer is rich in dendritic cells. In this study, M2 macrophages accumulated in CRCs of the elderly, and CD8+ cells, were able to predict a more favorable survival in stages UICC I-III of CRC, in contrast to metastatic (UICC IV) stages, in which CD4+ cells had the strongest impact on survival. Interestingly, immune infiltrates repopulated after rectal irradiation therapy. Taken together, this article opens timely new perspectives on the microenvironmental interaction of CRCs with immune cells and inflammatory components, extends ongoing efforts of additional CRC classifications into particular immune phenotypes, and suggests putative clinical, diagnostic, and therapeutic conclusions. Focusing back at biological changes in CRC tumor cells in a further original article, our own group presents a systematic whole-genome analysis of epigenetic changes across structural and, in particular, microRNA (miR) genes [4] in CRC. We suggest methylation changes in CRC, as compared to the normal colon or rectum, to occur especially in open sea regions and islands, and found that protein coding genes, but in particular genes of miRs that have been shown to be important within CRC progression and metastasis pathways, harbor significant methylation changes in primary CRCs. This adds to our knowledge of miR-regulation in cancer, since, up to now, rather transcriptional and genetic mechanisms of miR-deregulation in cancer, (CRC) progression and metastasis have been elucidated extensively. In a further original article, the group by Ulrike Stein et al. [5] extends our mechanistic knowledge on Wnt/catenin signaling, which is essential in CRC cell invasion and metastasis. They demonstrate an exciting novel means of transcriptional cross-regulation of S100A4 (which is a pro-metastatic Wnt target) and DKK1 (which is a Wnt antagonist) that includes an S100A4-induced feedback loop, which sustains Wnt signaling and associated metastatic properties. Thus, a combined measurement of S100A4 and DKK1 might be powerful as a biomarker for a more precise identification of high-risk patients in precision medicine. Finally, Pezeshkian, Nobili, Mini, and coauthors provide a comprehensive and timely overview on the current status of matrix metalloproteinases (MMPs) in their differential functions for CRC carcinogenesis, the transition from precancerous lesions to tumors, and CRC progression [6].

The issue of tumor cell heterogeneity is certainly still one of the most unresolved problems we are facing when it comes to (colorectal) cancer individual diagnosis, prediction, risk classification, and therapy. Ideally, we would like to aim at scenarios and early diagnostic tools that enable us to predict, for every single patient, the likelihood to develop later progressive disease or metastasis, before this actually happens macroscopically. Therefore, methods to identify metastatically “dangerous” tumor cell clones in any primary tumor, or within single disseminated tumor cells that can be identified in, e.g., the blood of cancer patients, would open tremendous opportunities for a more individualized risk prediction and (preventive) therapy stratification. In an interdisciplinary consortium of molecular translational oncology researchers, optical physicists, and chemists, the groups of H.A. Wagenknecht, C. Cremer, and our own group recently succeeded in establishing a first-in-field approach for quantitative single-cell, single molecule localization microscopy (SMLM) at the nanoscale, within resected human CRC tissues [7]. We specifically show examples of changes in chromatin nanostructure and intracellular distribution of microRNAs between individual (cancer) cells in the resected CRC patient tissue context. Such methodologies have a high potential to enable single-cell differential diagnosis between (cancer) cell clones of different mechanistic capabilities within individual tissues and tumors of patients in the future, aiming at a broadening of current macroscopic clinical imaging methods by microscopic and molecular imaging. Along the same lines, more and more sophisticated methods to (systematically) analyze single circulating (tumor) cells, or CTCs, within individual patients have an equally powerful potential for precision medicine. In an exciting review, Klaus Pantel and his group [8] present an actual status of CTC diagnosis and circulating DNA in the blood of CRC patients, and discuss their still increasing potential as easily accessible liquid biopsy markers able to indicate, e.g., response or resistance to therapy, for the prediction, monitoring, and management of CRC. A specific example of CRC monitoring within a prospective clinical study is impressively demonstrated by C. Alix-Panabieres and her group, in her original article which contributed to this Special Issue [9]. These colleagues show that CTC kinetics, as evaluated by the novel EPISPOT assay, are able to predict prognosis and, most likely, therapy response before and in the course of treatment in metastatic CRC, in a prospective, multicenter study (COLOSPOT). Taken together, all of these articles suggest that tackling tumor cell heterogeneity and single-cell diagnosis have higher chances than ever to enter clinically relevant settings.

Two further articles of this Special Issue show actual clinical study data, illustrating how, up to now rather neglected clinical characteristics (age and gender), can have an impact on clinical courses and outcomes in CRC, and, potentially, response to therapy. During recent years, it has been observed that sporadic CRC has been increasing, also, in younger patients. In an attempt to address potential causes for this, in a large multinational cohort of over 2100 newly diagnosed CRC patients, Himbert et al., together with many colleagues involved in the ColoCare study, analyzed patient, demographic, and lifestyle characteristics, compared between age of onset younger than, or over, 50 years [10]. The results of this study will help in guiding further research on CRC, especially in younger patients with no evidence of hereditary disease components. In another clinical study, Schuster et al. explore the impact of gender on the sensitivity to radio-chemotherapy in advanced rectal cancer [11]. Although these colleagues could not detect a significant difference in response between male and female patients, it is still interesting that female patients experienced an increased deterioration in quality of life following radio-chemotherapy, an observation that needs to be taken into account for future studies and therapy design. Moreover, the data can build a ground for further (re-) translational research on gender differences, in the response or resistance to particular therapies in CRC.

Translational, and re-translational, research in CRC, and its success and impact for clinical consequences will, ultimately, also depend on the availability of the right models that are able to reflect the situation within a CRC patient as authentically as possible. Therefore, two articles in this special issue introduce novel, and timely, means of modelling colorectal cancer. A highly interesting article by Jens Hoffmann and his group [12] introduces powerful patient-derived xenograft (PDX) models that are excellently suited to model personalized treatment in CRC, within defined molecular human patient subgroups, the genomic/molecular characteristics of the tumors being analyzed by systems biology approaches. The article demonstrates how biomarkers, or biomarker panels, can be developed for single drugs, or drug combinations, within these CRC PDX models, or how, for example, alternative therapeutic strategies could be suggested in the individual case, depending on individual oncogenic pathway analysis. Finally, the original article by Katja Steiger and her group [13] introduces amazing similarities in morphology and molecular pathways between human and feline CRC, clearly inviting us to broaden our perspectives to other species of our planet, when attempting to understand, and conquer, human diseases such as CRC. I, personally, think that this is an article coming at the right time, given our actual global alert on the threats of climate change, the increasing extinction of whole species by humankind, and the several-fold overdone exploitation of our planet by the human species. Perhaps, also, this article can encourage us to be more modest and respectful to other species on Earth, since there might be a number of species, some of them maybe already extinguished by us, of whom we could learn a lot for our own life and diseases, such as cancer. With this, the article again builds the bridge to zur Hausen’s and de Villiers’s review on, up to now, undiscovered species, discussed at the beginning of this Editorial.

Taken together, with this Special Issue, “Colorectal Cancers: From Present Problems to Future Solutions”, we hope to present an exciting compilation of articles, by well-known international experts, which can deepen discussions and ideas amongst colleagues in all kinds of disciplines working at CRC and beyond. I hope it can encourage, and intensify, even more translational and interdisciplinary collaborations, aiming at an ultimate understanding of strategies to defeat, and prevent, colorectal cancer, its progression and metastasis, and all the suffering and death resulting from it.
